# Toxicity of Saffron Extracts on Cancer and Normal Cells: A Review Article

**DOI:** 10.31557/APJCP.2020.21.7.1867

**Published:** 2020-07

**Authors:** Masihollah Shakeri, Akbar Hashemi Tayer, Heshmatollah Shakeri, Abdolreza Sotoodeh Jahromi, Malihe Moradzadeh, Mohammad Hojjat-Farsangi

**Affiliations:** 1 *Research Center for Non-Communicable Disease, Jahrom University of Medical Sciences, Jahrom, Iran. *; 2 *Golestan Rheumatology Research Center, Golestan University of Medical Sciences, Gorgan, Iran. *; 3 *Department of Oncology-Pathology, Immune and Gene Therapy Lab, Cancer Center Karolinska (CCK), Karolinska University Hospital Solna and Karolinska Institute, Stockholm, Sweden. *

**Keywords:** Cancer, cytotoxicity, Saffron extract

## Abstract

**Background and Aim:**

Medicinal plants have played an important role in human health since the Stone Age. According to WHO, 80% of Asian and African people rely on traditional medicine and medicinal plants to conserve their health. Saffron has received much attention among the herbal compounds related to cancer treatment.

**Methods:**

This review aims to provide an overview of in-vitro and in-vivo evaluation molecule mechanism for anti-tumor activity, cancer preventing and protective effects of saffron extract. The review is based on the available data accessible in PubMed, Science Direct, Google Scholar, Magiran.ir, and SID.ir databases.

**Results:**

Saffron has selective toxic and preventive effects on cancerous cells and without adverse effects on normal cells and prevents tumor formation. Saffron appears to reduce the toxic effects of anticancer drugs. Saffron has toxicity effects when used in high amounts, which are far greater than those are used in human food culture.

**Conclusions:**

Considering the observed effects of saffron on the removal of cancer cells, saffron extract can be used in the treatment and prevention of cancer after confirmation in human clinical trials. According to the high IC_50_ of saffron extracts in normal cells, its toxicity against non-cancerous cells is low and its use is safe. Besides, the studies suggested the cytotoxic effects of saffron on some of the more cancers, including nervous system cancer and common cancers. Further studies are required to determine the effective dose and influence of mechanism of saffron in various animal type of cancers.

## Introduction

Malignancy is the second cause of death and one of the worldwide major public health with a significant incidence and mortality rates in the developing and developed countries (Madani et al., 2010b; Gezici and Şekeroğlu, 2019). 

About 18.1 million new cancer cases and 9.6 million cancer deaths happened worldwide in 2018 (Ferlay et al., 2019). Some types of cancers are preventable by making appropriate changes in the lifestyle, diet and physical activities (Madani et al., 2010a; Heitz et al., 2018).

Among the various categories of cancer treatment including surgery, radiotherapy, hormone therapy, chemotherapy, targeted therapy, and herbal therapy, phytotherapy has been developed a tremendously important strategy for cancer management in recent years, due to its advantages for the treatment and prevention (Sanaei et al., 2018; Gezici, 2019; Sanaei et al., 2020). Medicinal plants have played an important role in human health since the Stone Age (Halberstein, 2005). According to WHO, 80% of Asian and African people rely on traditional medicine and medicinal plants to conserve their health (Organization, 1978). 

Due to the adverse effects of conventional cancer treatment drugs (Kokhaei et al., 2016; Nurgali et al., 2018; Hain et al., 2019) and drug resistance (Khamisipour et al., 2016), the prevention or treatment of cancer with herbal compounds has recently been considered by many researchers (Qi et al., 2015; Sanaei et al., 2017a; Sanaei et al., 2017b; Lou et al., 2018; Hain et al., 2019) among which saffron has been received much attention (Bhandari, 2015; Gudarzi et al., 2015). 

Originally, the word saffron originates from the French word Safran which comes from the Latin word Safranum. The saffron plant is a part of the Iridaceae family, which is an ancient spice and a natural food dye that has been used for the treatment of various illnesses in the long human history (Small, 2016; Khanali et al., 2017). This spice possesses numerous beneficial properties and traditionally it has been used as an anti-asthmatic, antidepressant, anti-blood pressure, antioxidant, anti-inflammatory, anticonvulsant, and as an antitussive. Saffron has recently been received attention for its anticancer properties (Hoshyar and Mollaei, 2017). 

Three-hundreds tons of saffron is produced annually in the world, 90% of which is produced in Iran (Khanali et al., 2017). The most important part of saffron for nutritional or therapeutic purposes is its stigma. The therapeutic properties of saffron stigma are due to the presence of three major secondary metabolites. They include: (i) Crocin (mono-glucosyl or di-glucosyl poly N-esters) and water-soluble derivatives that create the red colour of saffron; (ii) Picrocrocin (the glucoside precursor of safranal, responsible for the bitter taste of saffron); and (iii) Safranal (dicarboxylic acid precursor of crocin) that produces the scent of saffron (Hosseinzadeh et al., 2009; Hosseinzadeh et al., 2012). Low levels of thiamine and riboflavin are also present in saffron (Alonso et al., 2001; Azarabadi and Özdemir, 2018). Most of the beneficial and therapeutic properties of saffron are related to the antioxidant and anti-inflammatory effects of saffron extract and its active components (Lecomte et al., 2017; Hosseini et al., 2018; Zeinali et al., 2019).

There are considerable studies that focused on extraction of saffron and its active constituents to prevent and treat cancer, but the exact mechanism of these effects has not been discovered. An herbal compound is used as a prophylactic or therapeutic agent for a disease, that has slight or no toxicity and has high efficiency, edibility, a well-known mechanism of action, and low price (Abdullaev, 2002; Lecomte et al., 2017; Mani and Natesan, 2018). 

This review aims to provide a brief overview of in-vitro and in-vivo evaluation of molecular mechanisms for anti-tumor activity, cancer-preventing and protective effects, and toxicity of saffron based on the available reports in the literature.

## Materials and Methods

*Methods*

In this study, a search was conducted to investigate relevant studies in PubMed, Science Direct, Google scholar, Magiran.ir, and SID.ir databases. 

Keywords searched in the databases were extracted from MESH in the PubMed database including Saffron, Neoplasm, Cancer, Toxicity, Cytotoxicity effect, Tumoricidal effect, Protective effect, and Tumor.

The research included in which total saffron extracts (not one of the specific saffron ingredients) were interventional studies on humans and animals (in-vivo) as well as laboratory studies on cancer cells (in-vitro) for the period between 1975-2020. However, no study was found which focus on human.

The articles in which saffron was studied in a mixture with other herbs, specific saffron ingredient, and or the studied saffron belonged to a particular strain were excluded.

## Results

The results of the investigation of experimentally cell culture (in-vitro) and animal (in-vivo) studies in the literature on saffron extracts were included. However, no in-vivo human study was found. 

*Anti-cancer effect of saffron*

*In-vitro anti-cancer effect of saffron*

The effect of ethanolic extract of saffron on lung tumor cells and normal lung fibroblasts showed that tumor cells were more sensitive than normal cells to the inhibitory effect of the saffron extract on DNA and RNA synthesis (Abdullaev and Frenkel, 1992). Also, saffron may directly target DNA sequences and modulate transcription (Bathaie et al., 2007). 

Saffron extract at concentrations of 20, 40 and 100 μg/ml applied for 24, 48 and 72 hours reduced the expression self-renewal genes such as OCT4, KLF, SOX2, NANOG, and nucleostemin in gastric adenocarcinoma tumor cell line (Akbarpoor et al., 2020).

The effect of ethanolic extract of saffron at concentrations of 200-2000 μg/ml on HeLa cell line and Hep G2 cell line showed that IC_50_ on these two cell lines were 800 and 950 μg/ml after 48 hours, respectively. Apoptotic and non-apoptotic functions played a role in this toxicity. In that study, Reactive Oxygen Species (ROS) has no role in these effects and significant inhibition of DNA and RNA synthesis (50%) was observed at 100-150 µg/ml concentrations but no protein synthesis inhibition was observed even at high concentrations (Tavakkol-Afshari et al., 2008). 

The anti-cancer effects of saffron can be partially chain reaction, interference of carotenoids with topoisomerase (Abdullaev, 2002; Lee and Park, 2003; Patel et al., 2017), cell proliferation inhibition, apoptosis induction (Samarghandian et al., 2013; Patel et al., 2017; Colapietro et al., 2019b), inhibition of cell cycle progression and cell growth (D’Alessandro et al., 2013; Colapietro et al., 2019b; Gezici, 2019), activating an intrinsic apoptotic (caspase) pathway (Amin et al., 2011; D’Alessandro et al., 2013; Samarghandian et al., 2013), lactate dehydrogenase activity (LDH), antioxidant activity, DNA fragmentation (Gezici, 2019), cell differentiation enhancement, tumor metabolism modulation, stimulation of cell-to-cell communication and immune modulation (Colapietro et al., 2019b), radical scavenging (Wang and DU, 2018), arresting cell cycle progression, and matrix metalloproteinase expression suppression (Khorasanchi et al., 2018).

Ethanolic extract of saffron had a selective cytotoxic effect on epithelial cells like hepatocellular carcinoma and HeLa cell line, while it had no toxicity on normal fibroblast cells in rats (Tavakkol-Afshari et al., 2008). Saffron extract inhibits the cell viability and cell growth of cancerous cells in a time and concentration-dependent manner (Gezici, 2019).

Different concentrations of saffron extract; i.e. 100, 200, 400, and 800 μg/ml for three days on adeno-carcinomic human alveolar cells (A549) increased apoptosis in a dose-dependent manner (Samarghandian et al., 2013). 

Saffron causes nonspecific T-cell proliferation in the presence of phytohemagglutinin, indicating that anti-tumor activity of saffron may also be facilitated immunologically (Nair et al., 1995). The saffron extract reduced cell growth in human acute lymphoblastic T-cell leukaemia (Jurkat cell line) (Makhlouf et al., 2016). Table 1 shows the results of in-vitro studies of the anti-cancer effect of saffron. 

*In-vivo anti-cancer effect of saffron*

Despite the following studies indicating the beneficial anti-cancer effects of saffron, it is worth noting that most in-vivo studies have focused on the components of saffron and little attention has been paid on the saffron itself (Liu, 2004).

The chemoprotective effects of a saffron extract are related to its regulatory effect on lipid peroxidation, antioxidant activity and detoxification (Premkumar et al., 2003). 

Oral intake of 100 mg/kg body weight (bw) of saffron extract up to 12 weeks delayed the emergence and progression of skin tumors due to papillomavirus in the rat. Also, the same treatment with saffron extract restricted the tumor formation in soft tissue sarcoma induced in the rat (Salomi et al., 1991). Oral treatment with methanolic extracts of saffron at a dose of 200 mg/kg bw increased the lifespan of albino rats with transplanted sarcoma cells (Nair et al., 1991a). 

Oral treatment of saffron extracts in rats increased β-carotene and vitamin-A serum levels (El Daly, 1998) and based on the anti-cancer property of β-carotene, the anti-cancer effect of saffron may depended on this function (Tarantilis et al., 1994). Encapsulation of saffron extract with liposomes improved its anti-cancer effects in oral administration than intravenous administration (Nair et al., 1992). Administration of the aqueous extract of saffron at concentrations of 100, 150, 175 mg/kg bw after 50 days, dose-dependently inhibited gastric adenoma progression in rats (Bathaie et al., 2013). Table 2 summarises the results of in-vivo studies of the anti-cancer effect of saffron. 

*Protective effects of saffron extract*

Few studies examined the harmful toxic effects of saffron on normal cells. Khavari et al. showed that aqueous saffron extract had no cytotoxicity on non-malignant cell (Khavari et al., 2015). Also, D’Alessandro et al. found that saffron extract possesses anticancer activities with no cytotoxic effect on normal and nonmalignant cells (D’Alessandro et al., 2013). Gezici´s research revealed that saffron extracts had much more inhibitory properties on cell growth and viability in cancerous cells than in non-malignant cells (Gezici, 2019). Abdullaev´s work showed that saffron extract was not toxic up to 1500 μg and has not mutagenic effects (Abdullaev et al., 2003). 

Saffron has protected endothelial cells from damages such as oxidative stress and apoptosis (Rahiman et al., 2018) and it has also protected neurons against ROS and glucose toxicity. Saffron was suggested as a drug for diabetic neuropathy treatment (Mousavi et al., 2010).

Pre-treatment with aqueous saffron extract at 20, 40, and 80 mg/kg bw in Swiss albino mice significantly decreased the genotoxicity of anti-cancer drugs (Premkumar et al., 2001). Oral administration of 50 mg/kg bw of the saffron extract significantly diminished the toxicity of cisplatin (CIS) (El Daly, 1998), Another study has reported that a low dose of 2 mg/kg bw saffron extracts prolonged lifespan in rats treated with CIS and reduced the drug side effects (Nair et al., 1991b). Saffron has good potential to alleviate the toxicity of CIS, including the nephrotoxicity (Wang and DU, 2018). Saffron stigma extract at higher dosage produces less cytotoxicity as compared to a standard chemotherapy drug, cyclophosphamide (CPH). On the other hand, methanolic stigma extract has cytoprotection in peripheral blood lymphocytes (Malla et al., 2018). Chahine et al. showed that saffron extracts could have cardio-protective effects against Doxorubicin and/or ischemia/reperfusion injury in pre and post-treatment in rabbit (Chahine and Chahine, 2020).

Another study revealed that a dose of 80 mg/kg bw alcoholic extract of saffron during 30 days reduced rifampin-induced hepatotoxicity in rats (Mohajeri et al., 2011). Some protective effects of the saffron extract are listed in Table 3. 

*Side-effects of Saffron*

According to the literature, saffron has very low toxicity effects (Nair et al., 1991a; Nair et al., 1995; Lucas et al., 2001; Abdullaev, 2002; Wichtl, 2004). Contradicting results on the adverse effects of saffron have been reported. 

According to a study by Schmidt et al., (2007), consuming more than 10 grams of saffron may stimulate absorption and adverse effects including decreased appetite, insomnia, nausea, vomiting, dizziness, and miscarriage. However, other studies reported no toxicity for the consumption of up to 4 g/day over several days, even in pregnant women (Melnyk et al., 2010). There are few available reports on allergies to saffron (Lucas et al., 2001). 

In-vivo studies in animals found very little or no toxicity of saffron and its components (Nair et al., 1991a; Nair et al., 1995; Karimi et al., 2001). A relative high ID50 equal to 20 g/kg bw of saffron (Wichtl, 2004), and a relatively high LD50 of oral consumption of saffron equal to 20.7 g/kg bw (Abdullaev, 2002) were found from in-vivo studies; hence researchers consider saffron to be safe for human consumption.

High intake of saffron in the range of 200–400 mg/day alters the biochemical and haematological parameters in the normal range in healthy adults that are not clinically important (Modaghegh et al., 2008). Another study showed that intraperitoneal injection of saffron extract at high doses of 0.35, 0.70, 1.05 g/kg bw to rats caused anaemia and hepato-renal toxicity (Mohajeri et al., 2009). The results of studies on harmful effects and toxicity of saffron in in-vivo studies are presented in Table 4. 

**Table 1:** Anti-Cancer Effect of Saffron from in-vitro Studies

Research	Target cell	Results
(Abdullaev and Frenkel, 1992)	A549 cells (derived from a lung tumor), WI-38 cells (normal lung fibroblasts) and VA-13 cells (WI-38 cells transformed in vitro by SV40 tumor virus)	Inhibited selectively DNA and RNA synthesis in malignant cells
(Abdullaev et al., 2003)	Malignant cells (HeLa, A-204 and HepG2) and normal human cells	Inhibited selectively proliferation in human malignant cells.
(Chryssanthi et al., 2007)	MCF-7 and MDA-MB-231 breast cancer cells	Inhibited cancer cell Proliferation
(Tavakkol-Afshari et al., 2008)	Cervical carcinoma (HeLa cells), Hepatocellular carcinoma (Hep G2), non-malignant cells (L929)	Induced selectively cell death in malignant cells
(Mousavi et al., 2009)	Breast cancer (MCF-7) cells	Induced proapoptotic effects
(Amin et al., 2011)	HepG2 cells	Inhibited nuclear factor-kappa B activation, increased cleavage of caspase-3, DNA damage, and cell cycle arrest
(Samarghandian et al., 2013)	Pulmonary Tumor cells (A549)	Induced apoptosis by activation of caspase pathways
(D’Alessandro et al., 2013)	Human Prostate Cancer (BPH-1 cell line)	Inhibited cell proliferation, progression, induced cell cycle arrest and apoptosis by caspase-dependent pathway
(Festuccia et al., 2014)	Prostate Cancer Cells (PC3 and 22rv1)	Modulated Metalloproteinases and Urokinase Expression/Activity
(Khavari et al., 2015)	Papilloma virus induced malignant TC-1 cells	Induced apoptosis
(Gezici, 2019)	A549, MCF-7 and HeLa human cancer cells	Induced DNA fragmentation, cytotoxicity, cell death, inhibited cancer cell growth
(Akbarpoor et al., 2020)	Adenocarcinoma tumor cell line (AGS)	Reduced the expression of OCT4, KLF, SOX2, NANOG, and Nucleostemin genes

**Table 2:** Anti-Cancer Effect of Saffron from in-vivo Studies

Research	Animal/ Dose/ Time	Tumor/Cancer type	Results
(Salomi et al., 1991)	Mouse/ 100 mg/kg bw/ 12 weeks	Papilloma tumor	Restricted tumor incidence to 10%
(Salomi et al., 1991)	Mouse/ 100 mg/kg bw/ 12 weeks	Soft tissue sarcoma	Restricted the tumor formation
(Nair et al., 1991a)	Albino rats/ 200 mg/kg bw/ 12 weeks	Transplanted sarcoma cells	Increased the lifespan
(Das et al., 2010)	Swiss albino mice/ 200 mg saffron per kg bw/ day/mouse for 6-12 weeks	DMBA-induced skin carcinoma	prevented or delayed angiogenesis and tumor progression
(Bathaie et al., 2013)	Rat/ 100, 150, 175 mg/kg bw/ 50 days	Chemically-induced gastric cancer	Inhibited dose dependently tumor progression
(Festuccia et al., 2014)	Athymic nude mice/ 300 mg/kg bw/ 5 days/week for 2 weeks	Xenografted Prostate Cancer Cells (PC3 and 22rv1)	Delayed the occurrence of tumor progression, inhibited of Cell Invasion, reduced tumor growth
(Fujimoto et al., 2019)	ApcMin/+ mice / saffron extract (0.1% and 0.5%) diet for 4 weeks	Intestinal polyps	Decreased the number of intestinal polyps in a concentration-dependent manner.
(Nezamdoost et al., 2020)	Female BALB/c mice/ 75 mg/kg bw/ saffron aqueous extract with combination of high-intensity interval training (HIIT) for 4 weeks	Subcutaneously implanted 4T1 breast cancer cells	Suppressed tumor growth

**Table 3:** Protective Effects of Saffron Extract from in-vivo Studies

Research	Animal/ Dose/ Time	Results
(El Daly, 1998)	Rat/ 50 mg/kg bw/ 5 days	Saffron extract + cysteine significantly reduced toxic effects of CIS
(Nair et al., 1991b)	Rat/2 mg/kg bw/day / 16 weeks	Reduced the CIS side effects and prolonged lifespan
(Premkumar et al., 2001)	Mouse/ 20, 40, 80 mg/kg bw/ 5 days pretreatment	pretreatment with saffron can significantly inhibit the genotoxicity of CIS, CPH, mitomycin C (MMC) and urethane (URE)
(Amin et al., 2011)	Rat/ 75-300 mg/kg bw/day / 22 weeks	Hepatoprotection from cancer via modulating oxidative damage and suppressing inflammatory response
(Mohajeri et al., 2011)	Rat/ 80 mg/kg bw/day 30 days	Reduced rifampin-induced hepatotoxicity
(Premkumar et al., 2006)	Mice/ (20, 40 and 80 mg/kg b.w.) / for five consecutive days prior to the administration of anti-tumor drugs	Pre-treatment with saffron significantly inhibited anti-tumor drugs induced cellular DNA damage (strand breaks) as revealed by decreased comet tail length, tail moment and percent DNA in the tail

**Table 4:** Saffron Side-Effects from in-vivo Studies

Research	Research type	Treatment	Route of administration	Dosage	Toxicity
(Abdullaev, 2002)	Animal	Saffron	Oral	20.7 g/kg body wt	Equal to LD50, non-toxic
(Schmidt et al., 2007)	Animal	Saffron	Intraperitoneal Injection	1.2-2.0 g/kg bw	Nausea, vomiting, diarrhea, Bleeding
(Modaghegh et al., 2008)	Human	Saffron	Oral	200-400 mg/day	May change some hematological and biochemical parameters in normal ranges
(Melnyk et al., 2010)	Human	Saffron	Oral	4 g/day	Non-toxic

## Discussion

The findings of this study demonstrated that saffron extract has the antitumor function, cancer-preventing effects, selective toxicity against cancer cells, and protection of normal cells against the toxicity of anticancer drugs with no cytotoxicity on a normal cell. A number of mechanisms have been proposed for the anticancer activity of saffron and its components (Salomi et al., 1991; Nair et al., 1995; Moradzadeh et al., 2018). Saffron extract exerts its anti-cancer effects via RNA and DNA synthesis inhibition (Abdullaev and Frenkel, 1992; Abdullaev, 1994; Abdullaev, 2002; Tavakkol-Afshari et al., 2008; Patel et al., 2017), apoptosis induction (Tavakkol-Afshari et al., 2008; Samarghandian et al., 2013; Patel et al., 2017; Colapietro et al., 2019), self-renewal genes expression reduction (Akbarpoor et al., 2020), topoisomerase inhibition (Abdullaev, 2002; Lee and Park, 2003; Patel et al., 2017; Wang and DU, 2018), cell proliferation inhibition (Samarghandian et al., 2013; Patel et al., 2017; Colapietro et al., 2019), immune modulation (Khorasanchi et al., 2018), DNA fragmentation (Gezici, 2019), Metalloproteinase and Urokinase modulation (Festuccia et al., 2014), and cell growth reduction (Salomi et al., 1991; Bathaie et al., 2013; Makhlouf et al., 2016). Cancerous cells are more sensitive to the inhibitory effect of saffron on DNA, RNA and protein synthesis than healthy cells (Abdullaev and Frenkel, 1992; Tavakkol-Afshari et al., 2008). 

Saffron is involved in tumor prevention and the protection of normal cells through scavenging free radicals (Abdullaev, 2002; Lee and Park, 2003; Patel et al., 2017; Rahiman et al., 2018; Wang and DU, 2018), LDH activity (Gezici, 2019), detoxification (Nair et al., 1991b; Nair et al., 1993; Nair et al., 1994; El Daly, 1998; Premkumar et al., 2001; Premkumar et al., 2003; Mousavi et al., 2010; Mohajeri et al., 2011), inhibition of angiogenesis (Das et al., 2010), inhibition of tumor invasion (Festuccia et al., 2014), tumor formation restriction (Salomi et al., 1991), and lipid peroxidation regulation (Premkumar et al., 2003). 

An important point is that the anticancer function of different saffron compounds increases synergistically (Liu, 2004; Tavakkol-Afshari et al., 2008; Makhlouf et al., 2016), so saffron extract might have more anticancer power than its compounds. 

Although some studies reported very low toxicity of saffron and its components (Nair et al., 1991a; Nair et al., 1995; KARIMI et al., 2001) and considering its safe consumption for human being (Wichtl, 2004), allergy to saffron (Lucas et al., 2001), anaemia, kidney and hepatotoxicity have also been reported in high doses of saffron extract consumption (Mohajeri et al., 2009). 

The remarkable point about the anti-cancer function of saffron is that saffron is selectively targeted cancer cells (Kennedy Jr et al., 1986; Loomis and Hayes, 1996).Saffron and its components have low toxicity and can be even considered nontoxic in oral consumption as the actual quantity of saffron used in daily food consumption is much less than the amount that causes complications such as nausea, vomiting, diarrhoea, bleeding, haematological changes, and hepatic and renal toxicity (Escribano et al., 1996; Garc-Olmo et al., 1999; Modaghegh et al., 2008; Mohajeri et al., 2009). Saffron possesses a potent anti-tumor property and has an efficacious and safe treatment (Khorasanchi et al., 2018). 

However, despite numerous studies on the anticancer properties of saffron extract, no human clinical trial has been conducted to define the anti-cancer effects of saffron or its components. 

However, human clinical trial studies have been conducted on the effects of the saffron extract on other diseases such as allergic asthma (Zilaee et al., 2019), resolution of inflammation in the metabolic syndrome (Kermani et al., 2017; Boskabady et al., 2020), mild/moderate age-related macular degeneration (Broadhead et al., 2019), diabetic maculopathy (Sepahi et al., 2018), improving sleep quality (Umigai et al., 2018), and depression (Jam et al., 2017; Kashani et al., 2018; Lopresti et al., 2018; Moazen-Zadeh et al., 2018). 

It is noted that non-Hodgkin lymphoma (NHL), placed as worldwide the 5th to 9th most common cancer (Miranda-Filho et al., 2019) and also incidences of Hodgkin Lymphoma (HL) is increasing (Zhou et al., 2019). However, no study was found about the effect of the saffron extract on these cancers. 

In conclusion, this study was made to discover possibility of using saffron as a natural medicine and in particular as anti-cancer drug. Saffron exerts selective toxicity on the cancerous cell by mechanisms including apoptosis, arresting cell cycle progression, tumor cell metabolism modulation, RNA and DNA synthesis inhibition, DNA fragmentation, LDH activity, antioxidant activity, and self-renewal genes expression reduction. In addition, it prevents tumor formation by reacting with and scavenging free radicals. 

Also, saffron has anti-cancer and cancer-preventive effects in animal models of cancer, especially in skin, sarcoma, and gastric cancers, which can be related to its antioxidant and apoptotic function in cancer cells. Saffron can also exert antioxidant effects with its vitamin A precursor function. Saffron appears to have protective effects and reduce the toxic effects of anti-cancer drugs.

Saffron has selective toxic and preventive effects on cancer cells but has no side effects on normal cells. Saffron has toxicity effects when used in high dosage, which are far greater than those used in human food culture. Determining the effective dose and effect mechanism of saffron in various animal model cancers requires further studies. 

Considering the observed effects of saffron on the removal of cancer cells, saffron extract can be used in the treatment and prevention of cancer after confirmation in human clinical trials. 

Generally, due to the high IC_50_ of saffron extracts in normal cells, their toxicity against non-cancerous cells is low and its use is safe. Besides, studies suggested the effects of saffron on some of the more malignant cancers, including nervous system cancer and common cancers.

According to saffron extract has protective effects against some medications proposing an inventive therapeutic approach to reduce medicinal toxicities and revealing the way for potential clinical applications. In addition, research works for evaluating the effects of saffron extracts of lymphomas are suggested. 

The lack of human clinical trials has made it difficult to extend the results of experimental studies on animals to humans and define the best and safest dosage for humans; thus human clinical trials studies in this field are recommended.
